# The development of facility standards for common outpatient procedures and implications for the context of abortion

**DOI:** 10.1186/s12913-018-3048-3

**Published:** 2018-03-27

**Authors:** Nancy F. Berglas, Sarah C. M. Roberts

**Affiliations:** Advancing New Standards in Reproductive Health, University of California, San Francisco, 1330 Broadway, Suite 1100, Oakland, CA 94612 USA

**Keywords:** Abortion, Outpatient procedures, Office-based surgery, Facility standards, Evidence-based policy

## Abstract

**Background:**

In recent years, an increasing number of states have enacted laws that impose specific requirements for facilities in which abortions are performed. In this study, we sought to understand the processes used to develop facility standards in the context of other, less politically charged areas of health care and consider implications for the context of abortion.

**Methods:**

We conducted key informant interviews with 20 clinicians and accreditation professionals involved in facility standards development for common outpatient procedures (endoscopy, gynecology, oral surgery, plastic surgery). We examined the motivations for and processes used in facility standards development, use of scientific evidence in standards development, and decision-making in the absence of evidence. Interview data were thematically coded and analyzed using an iterative approach.

**Results:**

In contrast to U.S. state laws that target abortion facilities, standards for other outpatient procedures are commonly set by committees of clinicians organized by professional associations or accreditation organizations. These committees seek to establish standards that ensure patient safety without placing unnecessary burden on clinicians in practice. They aim to create evidence-based standards but can be hampered by lack of relevant research. In the absence of research evidence, committees rely on their clinical expertise and sense of best practices in decision-making. According to respondents, considerations of potential harm (e.g., deeper levels of sedation, invasiveness), rather than the specific procedure, should prompt additional requirements.

**Conclusions:**

If facility standards in the context of abortion were developed through processes similar to other outpatient procedures, 1) professionals who perform the procedure would be involved in standards development and 2) in the absence of clear research evidence, the expertise of clinicians, and the guidelines and standards of other organizations, are used to describe a best practice standard of care.

**Electronic supplementary material:**

The online version of this article (10.1186/s12913-018-3048-3) contains supplementary material, which is available to authorized users.

## Background

An estimated 926,000 abortions take place annually in the U.S., making it a very common outpatient procedure [1]. Since legalization in the 1970s, abortion has largely been performed in office and clinic settings [1], with a demonstrated safety record meeting or exceeding those of other outpatient procedures [2, 3]. Recently, an increasing number of states have enacted laws that impose specific requirements for facilities in which abortions are performed [4]. These are commonly referred to as TRAP laws, as they enact “targeted regulations of abortion providers” that do not affect other procedures or facilities. These laws are promoted as safeguarding women’s health, despite the lack of data indicating a patient safety problem and increasing evidence that such laws decrease women’s ability to obtain abortions [5–7].

The Supreme Court’s 2016 *Whole Women’s Health v. Hellerstedt* decision on the constitutionality of a Texas law requiring that abortions be performed in ambulatory surgery centers (ASCs) and by physicians with admitting privileges at a local hospital has been the most visible indication of this trend [8]. The Court struck down these requirements, holding that the burdens imposed by an abortion restriction cannot outweigh its likely benefits; that the court must assess such burdens and benefits on the basis of credible scientific evidence; and that differential treatment of abortion and other medical procedures may provide evidence of a lack of likely benefit. This last point makes it highly relevant to describe what facility standards development looks like in the context of other, less politically charged outpatient procedures. That is, understanding how facility standards are typically developed for outpatient care can inform what the process might look like for abortion in the absence of differential treatment.

### The migration of procedures to outpatient care

The past decades have seen marked increases in the number and scope of procedures and surgeries performed in outpatient settings [9]. Advances in anesthesia and minimally invasive surgical techniques, in conjunction with concerns about rising costs, resulted in a migration of care out of the operating room, first to hospital-based outpatient departments and then to freestanding ASCs and office-based settings. As of 2006, more than 48 million surgical and nonsurgical procedures were being performed annually on an outpatient basis [9].

The transition of care to outpatient settings led to many discussions about patient safety. The Institute of Medicine’s reports *To Err is Human* (1999) and *Crossing the Quality Chasm* (2002) focused national attention on the preventable mistakes that occur in the health care system and the need for consistent, high quality care for all patients [10, 11]. These reports resulted in changes to ensure that hospitals were meeting standards of quality care, as seen through the adoption of new safety practices, stronger accreditation requirements, and federal funding for patient safety research [12, 13]. These actions were slower to take place in the growing sector of outpatient facilities not affiliated with hospitals. Research on patient safety in freestanding ASCs and offices was initially quite limited [14], and specific attention to these facilities was rare.

In response, actions were initiated to protect the safety of patients having surgical and nonsurgical procedures in outpatient facilities not affiliated with a hospital. Facility standards – that is, policies that dictate the licensing, accreditation, physical plant and/or operations of a facility – are set under the auspices of varied entities, including the Centers for Medicaid and Medicare Services, state legislatures and regulatory agencies, independent bodies that accredit health care facilities, and professional associations that provide guidance to their membership. The laws governing outpatient facilities vary considerably across states, with some mandating specific details about facility operations and others enacting more general requirements, such as requiring that facilities be accredited. The actions of professional associations toward the development of facility standards for outpatient procedures differ by specialty. Both the American College of Surgeons and American Society of Anesthesiologists have long published guidance for physicians performing procedures outside of the hospital [15, 16]. Other groups have followed suit, promoting guidelines specific to their members and procedures. Most offer recommendations or voluntary certification programs (e.g., [17, 18], and at least one mandates specific standards that are required for membership [19]. In all cases, state laws and regulations supersede professional recommendations when content differs.

### Purpose of the study

This study examines how facility standards have been developed for procedures commonly performed in outpatient settings. Specifically, we use key informant interviews to investigate the motivations for the development of facility standards across different procedures; processes used to develop standards; use of scientific evidence in developing standards; and decision-making in the absence of evidence. To our knowledge, the process of facility standards development has not been systematically investigated. The use of qualitative in-depth interviews with experts involved in facility standards development allows for a rich understanding of that process.

We then consider the implications of these results for the context of abortion. We identify lessons learned from less politicized areas of medicine that may be applicable to abortion, if professional associations or accrediting organizations were to develop facility standards through a process in line with how similar entities have done this work. This application is particularly relevant to current abortion policy, given the Supreme Court’s recent holding that differential treatment of abortion and other medical procedures in state law may indicate that a law is unconstitutional.

## Methods

### Study design

We focused the scope of the study on four procedures commonly performed in outpatient settings, specifically gastrointestinal endoscopy, gynecology, oral surgery/dentistry, and plastic surgery. These specialties include relatively low-risk procedures that are commonly performed in ASCs or offices, as well as procedures that have been targeted by state laws (i.e., plastic surgery). Additionally, there have been publications about what, if any, facility standards are appropriate for these procedures (e.g., [17, 19–21]), thus indicating that facility standards development has been a topic of interest across these fields. However, we found that these documents often did not describe the process of how facility standards were developed. To gain a rich understanding of this process, we conducted in-depth qualitative interviews with key informants who were involved in facility standards development for these procedures.

### Participants

A purposive sampling strategy identified respondents who met study criteria (i.e., were physicians, dentists or accreditation professionals who had been involved in developing facility standards for one of the select outpatient procedures) and thus we believed could provide rich responses to interview questions. We identified respondents based on their known involvement in patient safety committees of professional associations or standards development committees of accreditation organizations (e.g., named in published articles, listed on publicly available committee rosters) and contacted them by email, phone and/or fax. Respondents were sent an information sheet, approved by the institutional review board, which outlined the study aims and protocol. At the start of the interview, respondents were offered the opportunity to ask questions or decline to participate. During the interview, we asked respondents for suggestions of further contacts to identify additional potential respondents (i.e., snowball sampling). Recruitment was ended when questions had been comprehensively addressed and were revealing no new themes, and a balanced distribution of professional specialties in the sample was reached. A total of 39 potential respondents were approached over the study period. Twenty agreed to participate and completed the one-time interview, and 19 did not respond to requests. There appeared to be no differences in respondent characteristics between those who accepted vs. declined to participate.

### Study procedures

The authors are social scientists with doctoral-level training in public health and experience conducting qualitative research. The authors are based at a research center housed in a clinical department at the University of California, San Francisco School of Medicine. This affiliation was noted in communications with potential respondents. The first author (who approached and interviewed the respondents) had no interaction with any of the respondents prior to the study; the second author had previously worked with one respondent on an unrelated policy research study.

The authors developed a semi-structured interview guide that asked respondents to discuss whether there were established facility standards for their specialty, what entities established those standards, motivations for developing standards, processes used to set and amend standards, use of research evidence in developing standards, and decision-making when evidence is absent or in dispute. [See Additional file 1.] The interview focused on the development of facility standards and did not address guidelines for clinical practice. The interview guide did not include questions about abortion, but rather asked respondents to reflect on their own medical specialty. Questions were open-ended and modified over time to probe emerging themes. The interview guide was piloted in the first five interviews with respondents across medical specialties. The study protocol and interview questions were found to function well (i.e., yielded the information being sought). No significant changes were made, and all transcripts were included in the final sample.

The first author conducted all interviews by phone between November 2015 and February 2016. Respondents provided verbal consent prior to the start of the interview. Interviews lasted 30 to 45 min. Interviews were audio-recorded and transcribed, and field notes were taken at the end of each interview. Respondents received a $50 gift card in appreciation of their participation. The study protocol was approved by the institutional review board of the University of California, San Francisco.

### Analysis

The analysis relied on a modified grounded theory approach [22] that included deductive coding based on the primary research aims (i.e., motivations for facility standards development, processes used in standards development, and evidence use), as well as inductive coding of themes that emerged from the data. First, the two authors independently reviewed a set of interview transcripts and developed preliminary thematic codes, which were compared and revised through ongoing discussion and then consolidated into a codebook. The first author applied the codebook to the entire set of interview transcripts using Dedoose qualitative data management software (SocioCultural Research Consultants, 2016). The second author reviewed all transcripts and provided ongoing input on codebook application and analysis. The authors reviewed the coded data for thematic patterns, including frequency of codes, commonalities and differences across interviews, and comparisons across medical specialties. Throughout, memos were developed and discussed to guide the analysis.

## Results

### Description of sample

The interview sample comprised 20 physicians, dentists and accreditation professionals who had been involved in developing facility standards for one of the select outpatient procedures. Interviews were distributed across the specialties: endoscopy (4), gynecology (5), oral surgery/dentistry (4), plastic surgery (4), and accreditation (3). No other information was collected about the respondents.

### Thematic results

Despite differences by medical specialty in the migration of procedures from hospital to non-hospital-based outpatient settings, a number of cross-cutting themes emerged from the data. These were grouped as three main themes, with associated sub-themes. In presenting these results, we focus primarily on the commonalities identified across medical specialties and note distinctions as relevant.

#### Motivations for facility standards

##### Protection of the patient

Across specialties, the primary motivation voiced in favor of having facility standards was protection of the patient. Patient safety was described by respondents as “the whole driver,” “the bottom line,” and “paramount for what we do.” As a plastic surgeon described, the aim of developing facility standards is to protect the patient: “It came about in the interest of patient care, promoting patient care, developing standards to try to ensure the highest level of care for the patient.”

Multiple respondents explained that facility standards for outpatient procedures are often based on concerns that quality could diminish when procedures take place outside of the more regulated hospital or hospital-affiliated setting. They described the importance of quality being equivalent across facility types. For some, this was framed as patients having “a right” or “deserving” equal care regardless of setting. A few respondents asserted that, consequently, it is the obligation of the provider to make sure patients feel secure in their decision. They noted that patients should not be taking unnecessary risks by having procedures in an ASC or office, or even perceive differences that make them feel they are at greater risk.

##### Prevention of rare events

More specifically, the push for facility standards was motivated by a desire to encourage providers to have processes in place that prevent and respond to unusual events. Multiple respondents emphasized that morbidity and mortality risks are generally low for procedures performed in outpatient settings; however, such risks do exist and must be taken into consideration. As one gynecologist noted: “It's not so much doing the procedure, which is pretty straightforward. It's more what to do if things go wrong.” Others described facility standards as a mechanism that compels providers to consider the processes needed to create the safest experience for patients.

##### Response to public concerns

While the desire to protect patients was universally expressed, the trigger to move beyond attention to safety in one’s own practice to developing formal facility standards was often reactive. Multiple respondents referred to public concerns about the safety of outpatient procedures as a strong instigator for state government and professional associations to establish facility requirements**.** Both plastic surgeons and endoscopists described standards that were initiated in response to adverse events that became publicized. Media attention to these events brought outpatient safety issues to the forefront and was an impetus for action. Some respondents – across all specialties – questioned whether these publicized adverse events were an appropriate justification for new standards or an overreaction to isolated events that do not reflect an actual patient safety problem. As one oral surgeon noted, “Nowadays, when somebody gets an infection or something, that could just snowball and all of a sudden there’s a state law that affects everybody for some aberrant case.”

#### Processes of facility standards development

##### Considerations of clinicians in practice

For committees of providers involved in developing facility standards, of particular concern was ensuring that standards were reflective of and responsive to clinicians in practice. As one plastic surgeon described his work on an accreditation standards committee: “There [have] to be standards, but the standards should be reasonable and the standards should be flexible.” Other respondents similarly noted the importance of ensuring that the standards not be “some ivory tower document” and balancing “the onerousness of the standards and the actual procedure being performed.” A few mentioned that standards must be flexible enough to deal with geographic diversity, whether that was the distance from a rural clinic to a tertiary care facility or the limits on real estate in an urban center. They emphasized that it is the intent of a standard – e.g., that the facility has a means for dealing with emergency transfers to hospital care – that must be assured, rather than the exact specifications of how to meet that standard.

Other respondents expressed concerns about the promulgation of standards that “sound great” in committee, but do not consider the impact of clinical practice. They explained that committees “can go too crazy with this stuff.” A few questioned whether having too many standards without emphasizing which are the most important could have a negative impact on patient safety. A gynecologist noted: “When you put on layer after layer of requirements, you start going in the other direction, where the workload becomes so high that important things can get missed.”

Respondents voiced support for accreditation entities that revisit standards on an annual basis, including in their process a period of public comment that allows providers and accreditation surveyors to give input on how existing standards perform in practice and propose new standards that may be beneficial. As one gynecologist involved in an accreditation standards committee described:

“You do the best you can, given what seems like a reasonable approach… based on the data we have today. But, again, I emphasize today. Because tomorrow, we may have different data, and so standards need to be flexible. They’re dynamic. They aren’t written in stone.”

##### Concerns about the involvement of state agencies

Respondents expressed strong concerns about the involvement of state legislatures in defining outpatient facility standards. Some felt that legislatures “focus on the wrong things,” resulting in standards that are “nonsensical” or even “draconian.” They described situations where laws are enacted even if they are not likely to result in public benefit. In part, this reflected concerns about ensuring that facility standards keep pace with changes in research and practice. This was seen to be difficult for state laws which, as described by a gynecologist, “don’t allow for customization and don’t change easily.” Others expressed concerns about non-clinicians legislating the practice of medicine, including one endoscopist who stated:

“Legislators typically are publicly reactive to the needs of the voting public when, in fact, that may or may not be associated in reality with the problem at hand.”

Some respondents, however, voiced support for the enactment of general regulations – such as mandating accreditation for ASCs – if the details remained with the medical community. They supported partnerships between state agencies with professional associations in crafting regulatory documents and with accreditation organizations to conduct reviews.

##### Leadership of professional associations and accreditation organizations

The efforts of professional associations and accreditation organizations to develop facility standards were largely supported by respondents. While this likely reflects the respondents’ own involvement in these efforts, respondents highlighted a number of advantages of the processes used by these groups. They described both types of organizations as engaging multidisciplinary clinical experts to participate in a deliberative consensus process of seeking input, reviewing available research evidence, and incorporating their own clinical expertise. Each type of organization was seen as having its area of influence and expertise. Respondents described patient safety committees of professional associations as having the benefit of understanding of specific practice (e.g., the unique needs of an oral surgeon) and, while lacking “regulatory teeth,” offer targeted standards for particular procedures, training, and settings. They described accreditation organizations as developing standards to meet the needs of a range of specialties and settings by including core content required for all facilities (e.g. patient rights, infection control, quality improvement) and adjunct content based on the individual needs of a practice (e.g., anesthesia, surgical, or pharmacy services).

##### Clinical needs that prompt new standards

Across specialties, respondents emphasized that it is the use of deeper levels of anesthesia and greater invasiveness of a procedure that should prompt additional requirements. Many framed the need for facility standards as founded on a continuum of risk, based upon the invasiveness of the procedure, length of the procedure, overall health of the patient and, especially, level of sedation used. For respondents, standards should be established based on considerations of potential harm, rather than the procedure itself. As described by an oral surgeon, “It doesn’t matter what procedure they’re doing… Anesthesia is anesthesia.” In such situations, a plastic surgeon noted, “more is required of the facility to make sure the patient is safe.”

#### Evidence use and decision making

##### Engagement with evidence-based medicine

Respondents expressed strong interest and intent to base facility standards on research evidence, and some frustration with the limits of being able to accomplish this goal. Across all respondents, there was recognition of the movement toward evidence-based medicine. One plastic surgeon noted, “The trends in our journals, our meetings, and our presentations has shifted – very consciously shifted – to more methodical research design.” In developing facility standards as part of accreditation or professional association committees, respondents across specialties described their efforts to bring an evidence-based approach to the work. They differed in their estimation of their success. Some described their standards as “definitely evidence-based.” Others firmly disagreed, with one endoscopist describing his committee as “really shocked” at how few [standards] are evidence-based.

##### Challenges undertaking an evidence-based process

Respondents reported that engaging in a formal methodological review of research evidence in developing facility standards is uncommon, although many committees seek out research to inform their decision-making. There was considerable variation in the extent to which respondents indicated that research evidence was taken into account. A few (in plastic surgery and endoscopy, specifically) described a formal search process used throughout the committee work that involved searching the primary research literature, seeking out unpublished conference papers, and grading levels of evidence. For others, formal processes to identify and review of research evidence was “not something that is typically part of the process.” Literature reviews appeared more common for professional associations developing guidelines for clinical practice and less common for accreditation organizations considering standards for the facility itself.

As noted by respondents, this may reflect the fact that research evidence on the impact of facility operations and policies is limited, hampering committees’ ability to make fully evidence-based decisions. Respondents described their surprise with the lack of research evidence about effects of outpatient facility standards, with one endoscopist noting that “evidence is not being collected to answer the questions we want to deal with.” Respondents mentioned the challenges in conducting research studies that examine the impact of particular facility standards, and also expressed some questions of whether such studies are even necessary to inform what the standard should be. The endoscopist further noted,

“There may not be evidence to say that you should have a quality improvement program because nobody’s actually conducted a study to show that the implementation of a quality assurance program improves outcomes…. But you can’t say that the absence of evidence of benefit from a quality assurance program means that we will not adopt a quality assurance program until somebody’s done the study to prove it. That’s contrary to common sense.”

Some accreditation organizations have begun to collect and respond to internal quality assurance data from the practices they accredit. This may include tracking trends in compliance to the organization’s standards, but also collecting mandatory reports of adverse events and random case reviews. Respondents involved with accreditation organizations saw these data as having particular value as they reflect “actual cases and problems within facilities.” Additionally, they described the standards development process as a means for noting evidence gaps and identifying future research questions.

##### Decision-making in the absence of evidence

In the absence of research evidence, committees establishing facility standards rely on their members’ clinical expertise and sense of best practices. Even when respondents wanted their standards to be evidence-based, they were hampered by limits of the research available. Without clear and conclusive data, an endoscopist noted, “a lot of it is expert opinion that’s put together.”

Some respondents stated that reliance on clinical expertise was not problematic, as it reflected years of on-the-ground experience. In contrast, others described this reliance as the cause of poorly developed standards. As one gynecologist noted, “This happens in committees because where there isn’t evidence, there’s no lack of opinion.” Respondents described how the influence of a persuasive committee member, and the makeup of the committee overall, may have a great effect on the standards that are produced. As the gynecologist described: “It ultimately comes down to people sitting around a table.”

When decisions are beyond the expertise of the committee members, the word of other experts was relied on, both “to make sure it’s not just a knee-jerk response” and to not “reinvent the wheel.” As one endoscopist described, “We reach out to a number of our sister societies across the world… discussing with them what [information] they used, where they found the literature, or where they found some guidelines, to leverage them.” Most commonly, these sources included the ASA guidelines on office-based anesthesia, as well as groups with expertise in infection control and occupational safety and health.

## Discussion

We sought to understand how facility standards are established for procedures commonly performed in non-hospital-based outpatient settings, including the motivations for the development of standards, processes used to set standards, and use of research evidence in developing standards. Despite differences across medical specialties in the transition to outpatient care, we found common perspectives that form the basis for understanding facility standards development, as well as its placement within the current world of evidence-based medicine.

The responses of the experts participating in our interviews are mirrored in the published academic and clinical literature. Across specialties, the establishment of facility standards is said to be motivated to protect patient safety across different settings, although often triggered by adverse events that threaten public confidence [17, 19, 23]. While the concept of having standards for outpatient facilities is largely supported by respondents and the literature [17, 19, 23], the specific content of what standards should include and the mechanism for developing them are still under discussion. One noted area of concern for respondents was the direct involvement of state legislatures – seen as lacking medical expertise, an understanding of clinical practice, and a means for regularly updating standards – in defining specific facility standards. In contrast, both professional associations and accreditation organizations were recognized for their ability to create standards that meet the needs of various specialties and types of outpatient facilities. Some respondents considered it appropriate for state legislatures to require that facilities be accredited by an external body, which would set and maintain the standards.

Recent decades have witnessed an important movement toward evidence-based medicine, in which robust research evidence is sought to drive decisions about health care practice and policy [24]. However, as respondents described and has been noted in published guidelines and reports [17, 19], there is little research that directly examines the impact of specific facility standards. For outpatient facility standards to be truly evidence-based, more and new kinds of research would be needed. In the meantime, it seems more appropriate to consider “evidence-informed” facility standards as the goal. Evidence-informed policy and practice take into account the best available scientific evidence, as well as professional expertise, contextual factors, and patients’ values and preferences in decision-making [25].

We had two motivations in undertaking this study: first, to understand the processes of facility standards development for outpatient procedures generally, and second, to consider the implications of these findings for abortion care specifically. States have increasingly singled out abortion facilities with regulations, citing a need to protect the safety of patients [26]. Yet, abortion in outpatient settings has a safety record established over 40 years in hundreds of peer-reviewed articles [27, 28]. The rate of complications following abortion is low across study designs, populations, provider specialties, procedure types, and gestational ages [2, 3, 29]. Nonetheless, the process of developing standards – through state laws that single out abortion facilities – appears notably different than the processes used for other outpatient procedures.

If a need for new standards for abortion facilities were to be identified, the results of this study point to some steps that should be taken to align the process of standards development with that of other procedures. Facility standards would be:

- Developed by professional associations and accreditation organizations that engage providers in an ongoing consensus process of content development, implementation, monitoring and revision, with a goal of having standards be reasonable and flexible.
- Developed across outpatient procedures of similar complexity and risk, and would not single out abortion.
- Informed by the best scientific evidence and, in its absence, the expertise of those who perform the procedure.
- Informed by published guidelines and expertise of other health professional organizations, such as anesthesiologists and infection control experts.

Such a process could build on existing clinical guidelines, developed specifically for abortion providers, which already include recommendations on provider training, infection control, emergency procedures, and other facility-level policies [30–33].

### Limitations

This study has limitations. First, we selected the four specialties based on our review of existing documents that address the development of facility standards, as well as common comparisons made within the field of abortion. Representatives from other specialties may have provided different responses in interviews; however, in our review of the existing research literature, we did not find evidence of a different dialogue about patient safety among other medical specialties. Second, we recruited respondents based on their experience working on the issue of facility standards, and thus their ability to provide rich responses to our questions. Their particular perspective likely affects some results. For example, their sense of the motivations for having facility standards may not reflect the range of opinions among providers in practice. They may also be more in favor of having standards than other providers would be. Third, this study focused on the development of facility standards, and did not address questions of enforcement, such as facility inspections or consequences for lack of compliance. Future research should examine how enforcement of facility standards works across different contexts. Finally, we did not ask respondents directly about the application of facility standards development to abortion. All interpretations of the applicability of facility standards for other outpatient procedures to the context of abortion are those of the authors.

## Conclusions

The processes used to develop facility standards across other medical specialties contrast with approaches that have been used for abortion in that: 1) professionals who provide the procedures are involved in developing standards and 2) in the absence of clear research evidence, the expertise of clinicians, and the guidelines and standards of other organizations, would be used to describe a best practice standard of care. To treat abortion like other – less politically charged – outpatient procedures, future efforts to develop facility standards that may apply in the context of abortion would be based on the best available scientific evidence and would involve health care providers who perform the procedure in the development, monitoring, and revision of the specific content of such standards.

## Additional file

Additional file 1: Interview guide. (PDF 41 kb)

## Abbreviations

ASC: Ambulatory surgery center
TRAP: Targeted regulations of abortion providers
