# Supplementary material for: The development of facility standards for common outpatient procedures and implications for the context of abortion
Source: BMC Health Serv Res. 2018 Mar 27;18:212. doi: 10.1186/s12913-018-3048-3 (PMC5870078; doi:10.1186/s12913-018-3048-3)
Supplement: Supplementary file 1 — Interview guide. (PDF 41 kb) [file 12913_2018_3048_MOESM1_ESM.pdf]

***Study*: The development of facility standards for common outpatient procedures and implications for the context of abortion**

INTERVIEW GUIDE

Introduction:

For this study, we are interested in understanding how facility standards are typically developed for outpatient procedures such as <procedure>. We have been reviewing existing materials regarding safety and standards for <procedure>, but we believe we may learn more from talking to experts like you with experience in practice. I have some questions to guide me, but I see this as an open discussion about your work and understanding of facility standards.

Do you have any questions before we begin?

May I record our conversation?

Interviewer to mark: ❒ YES, conversation may be recorded.
❒ NO, conversation may not be recorded.

Questions:

1. To your knowledge, are there facility standards in place for <procedure>?

   For the purposes of this study, we define facility or provider standards broadly as including transfer arrangements, physical plant requirements, staff credentialing, and facility procedures (e.g., medication storage, sedation, medical waste). This study will not assess standards of clinical practice.

2. Could you describe these standards for me?

3. What body (or bodies) developed these standards (professional clinician associations, government bodies, other organizations)?

4. What reasons are typically given in favor of having standards for <procedure>?

5. What reasons (if any) are given against having standards?

6. I'd like you to walk me through the process of developing facility standards for <procedure>, as you remember it.
   Probe for individuals and organizations (including expertise, status, etc.).

   A. Who initiated the process of developing standards?

   B. What prompted the process?

   C. Who was involved in the process of developing standards?

   D. Who drove this process?

E. How did the group reach consensus about each standard or recommendation?

F. What types of research evidence were reviewed as part of the process?

- How was this evidence reviewed?

- How, if at all, was it incorporated into the standards?

- Was there any other research evidence that you thought/think should have been reviewed? If so, what was that?

G. What types of clinical expertise were incorporated in the process? How was this expertise incorporated?

H. What types of other professional expertise was incorporated in the process? What kind? And how was it incorporated?

I. In your opinion, what types of information are most compelling for standards development?

J. Would you describe the standards as evidence-based? As evidence-informed? Why is that?

K. In general, how is research valued relative to clinical or other types of expertise (e.g., public health, legislative) in your field?

7. In your opinion, what was the biggest success of the process of developing standards?

8. Was there anything you felt was not successful? Where did it fail?

9. In your opinion, for what outpatient procedures are facility standards needed?
Probes:
    - Which bodies should set those standards (government, professional associations, others)?
    - Are there any that should go through a different process?
    - Are there any that should be set by state (law)?
    - Why might standards be needed for that procedure but not for others?

Conclusion:

10. Is there anything we did not cover today that you think might be useful for our study?

11. SAMPLING: Is there anyone else in your field we should talk to about this topic?

12. IDENTIFICATION: We may want to include your name or professional affiliation in our final papers and presentations, to give further context to our findings. If you prefer that your responses remain anonymous, we will not link your name to any information or opinions that you share. However, because we are interviewing a small number of people, it may be possible for others to guess or assume who you are based on your responses to questions and our description of your expertise. Given what we have discussed today, are you comfortable with our using your name or affiliation?

Interviewer to mark: ❒ YES, responses may be identified.
❒ NO, responses may not be identified.
